# Dithiooxamide Modified Glassy Carbon Electrode for the Studies of Non-Aqueous Media: Electrochemical Behaviors of Quercetin on the Electrode Surface

**DOI:** 10.3390/s120403916

**Published:** 2012-03-26

**Authors:** Ayşen Demir Mülazımoğlu, Ecir Yılmaz, İbrahim Ender Mülazımoğlu

**Affiliations:** Department of Chemistry, Ahmet Keleşoğlu Education Faculty, Konya University, 42099 Konya, Turkey; E-Mails: admulazimoglu@gmail.com (A.D.M.); eciryilmaz@gmail.com (E.Y.)

**Keywords:** electrochemical surface modification, electrochemical surface characterization, scanning electron microscopy, quercetin, dithiooxamide

## Abstract

Electrochemical oxidation of quercetin, as an important biological molecule, has been studied in non-aqueous media using cyclic voltammetry, electrochemical impedance spectroscopy and scanning electron microscopy. To investigate the electrochemical properties of quercetin, an important flavonoid derivative, on a different surface, a new glassy carbon electrode has been developed using dithiooxamide as modifier in non-aqueous media. The surface modification of glassy carbon electrode has been performed within the 0.0 mV and +800 mV potential range with 20 cycles using 1 mM dithioxamide solution in acetonitrile. However, the modification of quercetin to both bare glassy carbon and dithiooxamide modified glassy carbon electrode surface was carried out in a wide +300 mV and +2,800 mV potential range with 10 cycles. Following the modification process, cyclic voltammetry has been used for the surface characterization in aqueous and non-aqueous media whereas electrochemical impedance spectroscopy has been used in aqueous media. Scanning electron microscopy has also been used to support the surface analysis. The obtained data from the characterization and modification studies of dithioxamide modified and quercetin grafted glassy carbon electrode showed that the developed electrode can be used for the quantitative determination of quercetin and antioxidant capacity determination as a chemical sensor electrode.

## 1. Introduction

Electrochemical and spectroelectrochemical measurements leading to the determination of kinetic parameters for antioxidants (e.g., redox potential, number of electrons transferred, electrode reaction rate constant, *etc.*), are very relevant, not only for evaluating the antioxidative abilities of flavonoids [1], but also for understanding their reaction mechanisms. The half-wave potential (E_1/2_) is a useful parameter for supplying information on the scavenging activity of the flavonoids. This has been rationalized on the basis that both electrochemical oxidation and hydrogen-donating free radical scavenging involve the breaking of the same phenolic bond between oxygen and hydrogen, producing the phenoxy radical and hydrogen, in an electron and proton transfer reaction. Thus a flavonoid which has a low value of E_1/2_ is a good scavenger [2,3].

Electrochemical studies revealed general trends in the electron-donating abilities of flavonoids [4]. The reduction potentials of flavonoids depend strongly on the electron donating properties of the substituents in the ring B [5], and on the ring B, the most oxidizable phenolic group is the more basic site [4]. The pKa values were assigned to ring A and ring B following reference [6]. The mechanism of phenolic compound action as antioxidants seems to involve the ability of phenols to scavenge radicals by an H-atom or electron transfer process by which the phenol is converted into a phenoxyl radical. The ease of oxidation of the phenol is of importance for its effectiveness as an antioxidant [7].

**Figure 1.**
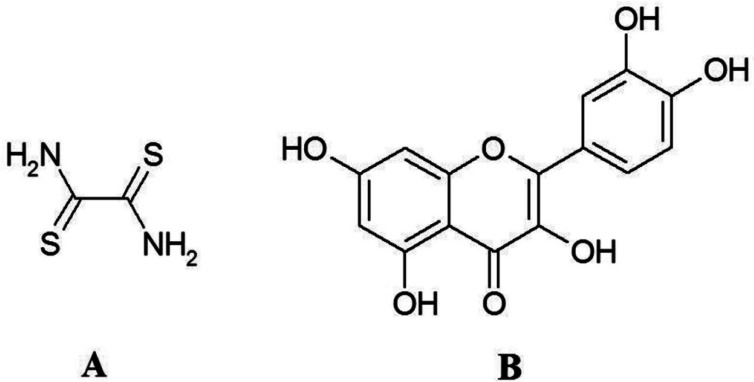
Structural features of (**A**) dithiooxamide (DTO) and (**B**) quercetin (QR).

Quercetin is one of the most abundant plant-derived polyphenols and is widely consumed with a human diet [8]. Most flavonoid molecules have the same structure as quercetin, except that they have a specific sugar molecule in place of one of quercetin hydroxyl groups on the C ring, which dramatically changes the activity of the molecule. Quercetin is the 3,3′,4′,5,7-pentahydroxyflavone and the corresponding chemical structure is shown in Figure 1(B). The literature on the electrochemistry of quercetin is limited [9–21]. However, Zhu *et al.* [20] investigated the electrochemical behavior of the interaction of quercetin with DNA. Nematollahi and Malakzadeh [15] described the electrooxidation of quercetin in the absence and presence of benzensulfonic acid and 4-toluenesulfonic acid as nucleophiles. Liu and Guo [21] studied the interaction of flavonoid, quercetin with organized molecular assemblies of nonionic surfactant. On account of these properties it is widely used as beneficial food supplement that is recommended for prevention and suppression of many diseases associated with oxidative stress. It has also become evident that antioxidant therapy may lead to adverse effects, as the ability of quercetin and other flavonoids to cause damage to cellular macromolecules and induce formation of reactive oxygen species has been discovered [22–24]. The most accepted explanation of the adverse effects is the formation of flavonoid oxidation products [25,26]. The main intermediates formed during oxidation are semiquinone and reactive electrophilic o-quinones.

The sulfonamide group in unsaturated nitrogen-containing ligands results in a decrease of the σ-donating ability of the nitrogen atom lone pair and an increase of the π-acceptor properties of the chelating bidentate fragment [27].

Dithiooxamide (rubeanic acid, NH_2_C(=S)C(=S)NH_2_), Figure 1(A)), is the sulfur analog of oxamide. Existing S and NH_2_ groups in this ligand offers its strong tendency to adsorption on the soft metal surfaces such as mercury. Dithiooxamide has similar molecular structure to thiourea (thiooxamide) molecule and it includes more S donor atoms. Dithiooxamide monomers were studied by FTIR spectroscopy combined with the low-temperature matrix-isolation technique. The most stable dithione-diamino tautomer of the compound was exclusively observed in argon matrices immediately after deposition [28].

Electrode modification, with an important part in electrochemical studies, has been extensively used for the last decade. As a matter of fact, these modified electrodes have come into prominence in determination of organic and inorganic species, especially in that of trace amounts in natural samples.

As the surface of an electrode can be prepared in an appropriate medium and under the optimum preparation conditions through electrochemical oxidation or reduction [29–31], modified electrodes can be physically prepared as in carbon paste electrodes [32]. Although electrode modification is done widely in the aqueous media [33–36], recent studies in non-aqueous media [37,38] are also available. Because the determinations in studies are usually made in the aqueous media, which is thought to be more stable, the modification processes in aqueous media are preferred. However, using the sensor electrodes obtained after modification, the determinations of some species in natural samples, phenolic compounds [32,37,39], metals [40–42] and *etc.* can also be made in the aqueous media.

In this study, the electrochemical mechanism of oxidation of quercetin dihydrate was investigated, for a wide range of non-aqueous solution conditions onto the dithiooxamide modified GC electrode surface, using CV, EIS and SEM techniques. Information on the mechanism of quercetin oxidation obtained from results at studies may play a crucial role in understanding its antioxidant activity.

## 2. Experimental Section

### 2.1. Chemicals and Solutions

DTO and QR were of analytical-reagent grade supplied from Sigma-Aldrich. The other chemicals used for electrochemical experiments were purchased from Riedel and Sigma-Aldrich. DTO and QR solutions in non-aqueous media used in modification were prepared 1 mM concentration in 100 mM tetrabutylammonium tetrafluoroborate (TBATFB) (in acetonitrile (MeCN)). Britton-Robinson (BR) buffer solution, pH 2, which was prepared from H_3_PO_4_ + CH_3_COOH + H_3_BO_3_ according to preparation conditions in the literatures [32,37] and then adjusting of pH by addition of 0.2 M or 1 M NaOH.

### 2.2. Electrochemical Equipment and Apparatus

A traditional three-electrode cell system was used in all electrochemical and spectroelectrochemical experiments. In experiments, GAMRY Reference PCI4/750 series Potentiostat/Galvanostat/ZRA from GAMRY Instruments (Warminster, PA, USA) electrochemical analyzer with BAS (Bioanalytical Systems, West Lafayette, IN, USA) Model MF-2012 and Tokai GC-20 GC electrodes were used. Ag/Ag^+^ (10 mM AgNO_3_) (BAS Model MF-2042) for non-aqueous media and a Ag/AgCl/3 M KCl (BAS Model MF-2063) for aqueous media were used as reference electrodes. Pt wire (BAS Model MW-1032) was used as counter electrode. Jenway 3010 pH meter was used for the measurement of pH values. The cyclic voltammetry (CV) technique was applied with PHE200 Physical Electrochemistry software, electrochemical impedance spectroscopy (EIS) was applied with EIS300 Electrochemical Impedance Spectroscopy software. The morphology of DTO and QR films on glassy carbon (GC) electrode surface was investigated by using scanning electron microscopy (SEM, Carl Zeiss LS10 Series SEM, Kansas City, MO, USA).

### 2.3. Electrode Preparation and Modification

The GC electrodes were prepared for the experiments by polishing to achieve a mirror-like appearance, first with fine wet emery papers (grain size 4000) and then with 1.0 μm and 0.3 μm alumina slurry on micro cloth pads (Buehler, Lake Bluff, IL, USA). After the initial polishing, the GC electrodes were resurfaced with 0.05 μm alumina slurry. First, in the following order, the GC electrodes were sonicated both in water, and in 1:1 (v/v) isopropyl alcohol (IPA) and MeCN (IPA + MeCN) mixture for 10 min [32,37].

The electrochemical modification of the GC electrodes were performed with 1 mM DTO in MeCN containing 100 mM TBATFB as supporting electrolyte in non-aqueous media from 0.0 mV to +800 mV potential ranges using 100 mV·s^−1^ sweep rate with 20 cycles. After the modification of the GC electrode, the surface of obtained dithiooxamide modified GC (DTO/GC) electrode was washed in order to remove all impurities from the electrode surface and then it was used for other investigations described in this study.

1 mM QR was prepared in 100 mM TBATFB (in MeCN). To investigate the electrochemical and spectrochemical features of QR on bare GC and DTO modified GC electrode surfaces, QR was bound to both surfaces applying potential in the range from +300 mV to +2,800 mV with 10 cycles at 100 mV·s^−1^ scanning rate.

### 2.4. Electrode Characterization

The modified electrode surface characterizations after the modification process were carried out by CV, EIS and SEM. The characterization with CV technique was performed in the presence of: (a) K_3_[Fe(CN)_6_] (Fe(CN)_6_^3−^) in BR buffer, pH 2.0, and (b) 1.0 mM of ferrocene in MeCN containing 100 mM TBATFB as redox active probes. In the characterizations with CV, ferrocene solution was carried out the potential range from −200 mV to +500 mV in non-aqueous media and Fe(CN)_6_^3−^ in BR buffer solution, pH 2, was performed the potential range from +600 mV to −100 mV in aqueous media at sweep rate of 100 mV·s^−1^. The cyclic voltammograms of modified electrodes were compared with cyclic voltammograms of the bare GC electrode. The characterization with EIS technique was carried out with a Gamry Reference PCI4/750 potentiostat by EIS 300 software. For the EIS measurements performed in 100 mM KCl containing 1.0mM equiv.-molar ratio of Fe(CN)_6_^3−^/Fe(CN)_6_^4−^, a sine wave potential alteration at 5 mV amplitude superimposed on a formal potential of the redox probe of 220 mV was applied, a wide frequency range from 100.000 Hz to 0.05 Hz was scanned and the Nyquist plots were recorded. The Nyquist plots of modified electrodes were then compared with the EIS data of the bare GC electrode. All potentials are referenced *vs.* Ag/AgCl/(3 M KCl) for aqueous media and Ag/Ag^+^/(10 mM AgNO_3_) for non-aqueous media. The morphology of DTO and DTO-QR films on GC electrode was investigated by SEM.

## 3. Results and Discussion

### 3.1. Modification of GC Electrodes

From our earlier studies [9–11,32,37,43–45] showed that performed electrode modification of amine containing groups and flavonoid derivatives becomes more stable in non-aqueous media. Therefore we have used non-aqueous medium for the modification of DTO onto the GC electrode surface and QR binding to the modified surface in this study. The electrochemical and spectrochemical properties of QR on DTO modified GC (DTO/GC) electrode surface was studied in non-aqueous media. The results from CV, EIS and SEM measurements were given together with bare GC electrode surface in the related sections.

**Figure 2.**
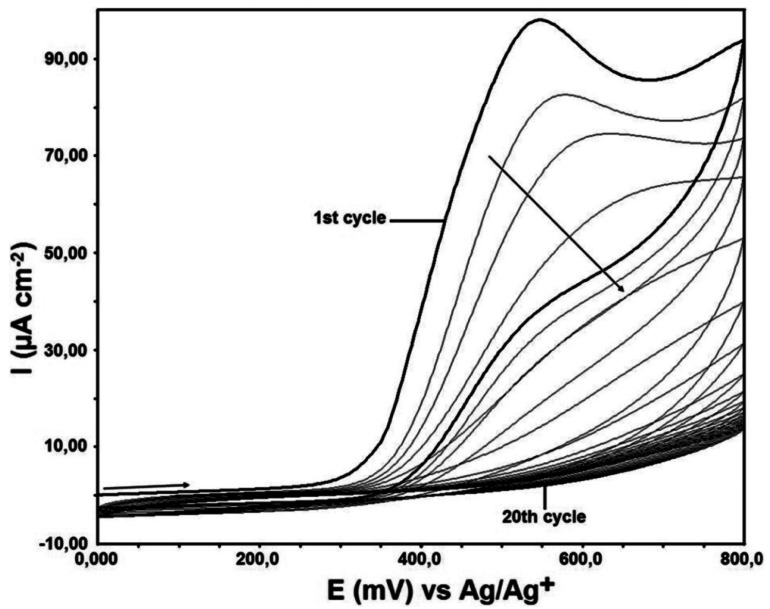
Cyclic voltammogram of 1 mM DTO in 100 mM TBATFB (in MeCN) *vs.* Ag/Ag^+^/(10 mM AgNO_3_). Potential sweep rate is 100 mV·s^−1^.

For this purpose, the GC electrode surface was modified by using a solution of 1 mM DTO, in a non-aqueous media, 100 mM TBATFB (in MeCN). Modification was being done at the potential range from +0.0 mV to +800 mV. Modification process was done 20 cycles and at the sweeping rate of 100 mV·s^−1^ (Figure 2).

**Figure 3.**
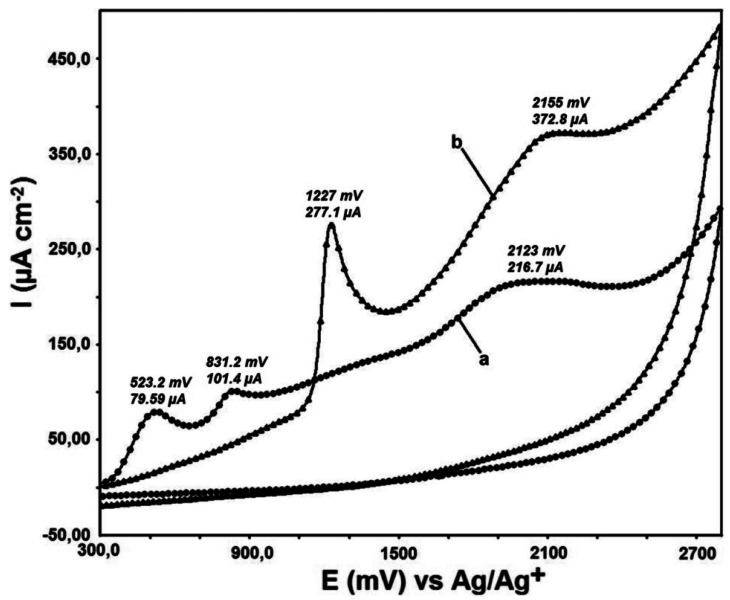
Overlaying of surface voltammograms of the first cycle (**a**) QR modification on bare GC and (**b**) QR modification on DTO/GC surface from +300 mV to +2,800 mV, 10 cycles, 100 mV·s^−1^ sweeping rate.

QR modification onto the bare GC and DTO/GC electrode surfaces was performed by applying a potential from +300 mV to +2,800 mV with 10 cycles at 100 mV s^−1^ scanning rate (Figure 3). Peak potential and peak current values are shown on the overlayed figure.

While there are two peaks at 523.2 mV and 831.2 mV for QR binding to the bare GC electrode, there is only a sharp peak at 1,227 mV for QR binding to the DTO modified GC electrode surface. The QR binding peaks are at 2,123 mV and 2,155 mV for bare GC and DTO/GC electrode surfaces respectively. The binding peaks are observed at closely same voltage. A sharp peak observed at 1,227 mV is a sign of sensitivity of this electrode towards QR molecule. This feature can be used for the determination of QR by utilizing DTO/GC electrode.

### 3.2. Characterization of Electrode Surfaces

**Figure 4.**
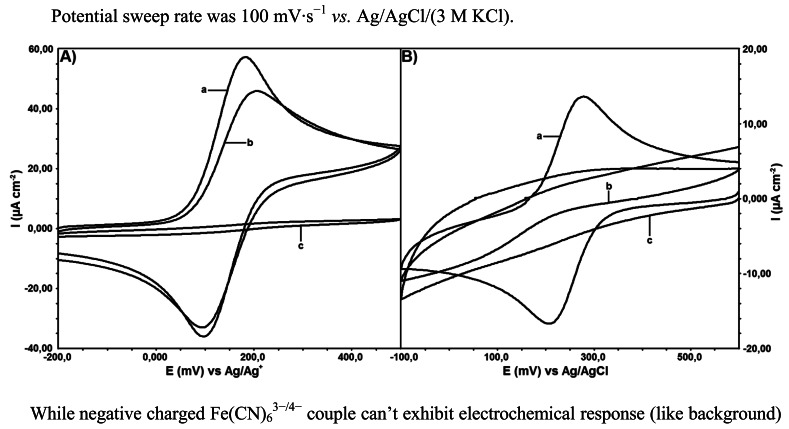
Cyclic voltammograms of (**A**) 1.0 mM ferrocene in MeCN with 100 mM of TBATFB on the (**a**) bare GC, (**b**) DTO/GC and (**c**) DTO/GC-QR electrodes. Potential sweep rate was 100 mV·s^−1^ *vs.* Ag/Ag^+^ (10 mM AgNO_3_); (**B**) 1.0 mM Fe(CN)_6_^3−^ in BR buffer solution, pH 2.0, on the (**a**) bare GC, (**b**) DTO/GC and (**c**) DTO/GC-QR electrodes. Potential sweep rate was 100 mV·s^−1^ *vs.* Ag/AgCl/(3 M KCl).

As the surface characterization processes after modifications were performed electrochemically by using ferrocene redox probe in non-aqueous media and Fe(CN)^3−^ in aqueous media with CV, spectroscopically they were performed with EIS using a mixture of Fe(CN_6_)^3−^/Fe(CN_6_)^4−^ redox probe and SEM. Electrochemical capability of modified GC electrode was shown in Figure 4 using two different redox couples: ferrocene (neutral)/ferrocenium (positive charged) in non-aqueous solutions (Figure 4(A)) and Fe(CN)_6_^3−/4−^ (negative charged) in aqueous solutions.

While negative charged Fe(CN)_6_^3−/4−^ couple can't exhibit electrochemical response (like background) in aqueous solution (Figure 4(B)), ferrocene/ferrocenium couple show electrochemical activity on modified electrode in non-aqueous solutions.

Especially the reduction current of ferrocenium ion can't be completely ignored, compared to the bare GC electrode results. The positive charge of ferrocinium ion must be considered for conclusion. Surface charge density might affect the electrochemical behavior of modified electrodes. Oxidation-reduction of redox probe species occurs with rapid redox kinetics at the bare electrodes as indicated by the peak shapes of the voltammograms.

No peak is observed on the voltammograms of the surface characterization tests performed by ferrocene and ferricyanide on electroinactive surfaces. In the current research, bare GC and DTO/GC surfaces are electroactive, whereas DTO/GC-QR surface is electroinactive. This result is an expected one and an evidence for binding of QR molecule on DTO/GC electrode surface. Similar result can be seen in the Nyquist test done by EIS. As a conclusion bare GC and DTO/GC electrode surface does not show any resistance to the electron transfer but DTO/GC-QR electrode surface shows resistance to the electron transfer up to 10 kΩ.

According to the data obtained from the characterization procedures, an interpretation was done over the fact that DTO and QR molecules in non-aqueous media are one or multi-layered on the surface of the GC electrode.

**Figure 5.**
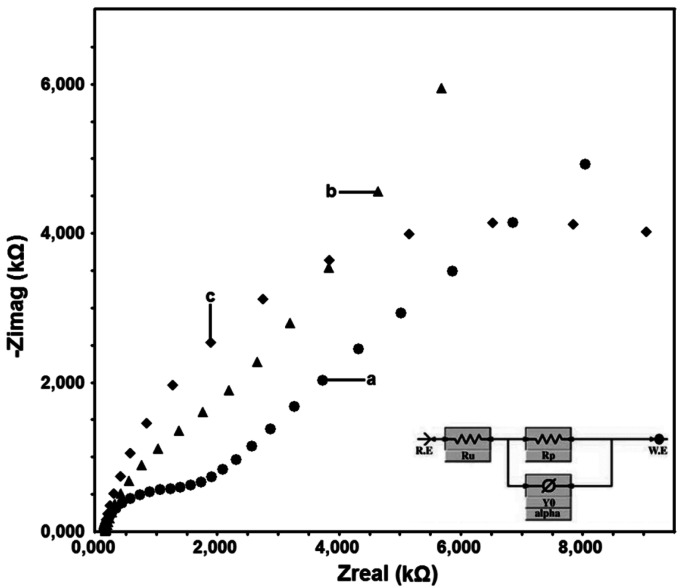
Nyquist plots of 1 mM of Fe(CN)_6_^3−^/Fe(CN)_6_^4−^ in 0.1 M of KCl of bare GC (**a**), DTO/GC (**b**) and DTO/GC-QR electrodes (**c**). Frequency range is from 100.000 Hz to 0.05 Hz the modulation amplitude is 5 mV. Inset: Equivalent circuit applied for calculations.

The surfaces obtained after characterization processes were evaluated and then it was presupposed that the surface obtained after the modification in non-aqueous media was suitable for the application. This case was supported by the results of the EIS obtained by using 5 mV potential and a mixture of 1 mM Fe(CN_6_)^3−^/Fe(CN_6_)^4−^ at 100.000 Hz and 0.05 Hz. The EIS graphs obtained after modifications are given in Figure 5.

The bare GC surface allows the electron transfer because it is sensitive to the Fe(CN)_6_^3−/4−^ redox probe. Since the DTO/GC-QR electrode surface is occupied by QR molecules, the surface does not allow for the electron transfer and shows resistance to some degree.

**Figure 6.**
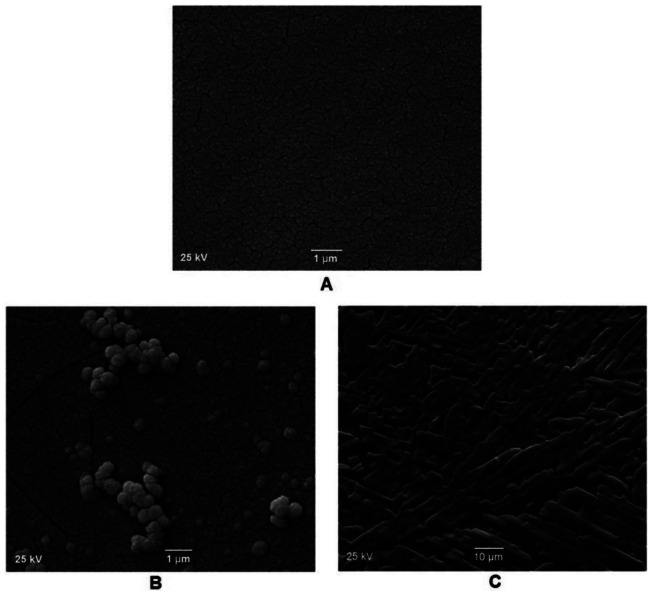
SEM images of (**A**) bare GC, (**B**) DTO/GC and (**C**) DTO/GC-QR.

In addition to CV and EIS measurements, scanning electron microscopy (SEM) was applied for characterization of bare GC, DTO/GC and DTO/GC-QR layers electrodeposited on the GC electrode surfaces. SEM images of an electrodeposited DTO and DTO-QR films on GC electrode are shown. Figure 6 shows the SEM images for the surface of bare (Figure 6(A)) and modified (Figure 6(B) and Figure 6(C)) GC electrodes. As shown, the distribution of DTO nanoparticles on the amorphous GC electrode surface is low. In addition to small submicron particles distributed on the surface, large agglomerated particles are also observed on the image (top right). However, the distribution of DTO-QR on the amorphous GC electrode surface is high. The inset of this figure shows SEM image with higher magnification for the same sample (the scale bar is about 1–10 μm).

### 3.3. Grafting and Oxidation Processes of QR onto the GC Electrode Surfaces

QR has two different pharmacophores, the catechol group in ring B and the three hydroxyl groups in rings A and C. The activity of the three OH groups is enhanced by an electron donating effect of the hydroxyl groups at positions 5 and 7. The hydroxyl groups in ring B are electron-donating and stabilize active intermediates, and the C-3 hydroxyl group can also form intermolecular hydrogen bonds with the oxygen at C-4, also stabilizing active intermediates.

QR oxidation processes proceed in a cascade mechanism and are related with the catechol groups in ring B and the three hydroxyl groups in rings A and C which all present electroactivity, and the oxidation potentials are identified with the experimental peaks 1–3 described previously. The oxidation of the catechol moiety, 3′,4′-dihydroxy electron-donating groups at ring B, occurs first at very low positive potentials corresponding to peak 1, and is a two electron two proton irreversible reaction.

The hydroxyl group at position 3 at ring C is oxidized afterwards, corresponding to peak 2, was shown to undergo an irreversible oxidation reaction, and is a very small peak because the hydroxyl group can also form an intermolecular hydrogen bond with the oxygen at position 4 at ring C [2,21,32,46–48].

**Figure 7.**
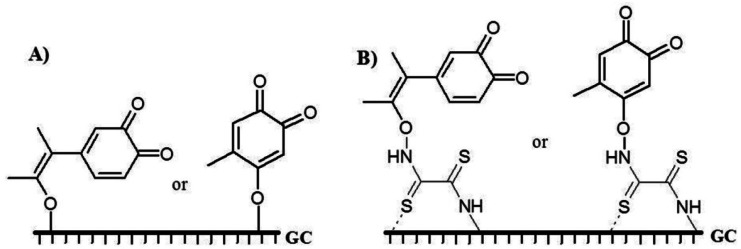
Grafting QR onto the (**A**) bare GC and (**B**) DTO/GC electrode surfaces.

Peak 3 appeared at very high potential. This peak was related to the binding of QR to the GC electrode surface through C–O–C or C–O–N binding. Possible mechanisms of binding of QR to the bare GC and DTO/GC electrode surface were given in Figure 7 considering the obtained results and the related published articles before [49–54].

After all these studies, GC electrode surface was modified by DTO molecule as monolayer through amine oxidation in non-aqueous medium. To find out the sensitivity of modified surface towards flavonoid derivatives. For this purpose, most known and high antioxidant capacity QR, a flavonoid derivative, was used. This study showed that DTO/GC electrode surface is very sensitive towards QR molecule. Using this feature, QR can be quantitatively determined in various plants by the new developed sensor electrode. Antioxidant capacity can also be determined using this developed electrode.

## 4. Conclusions

This study precedes studies initiated to find out the ability of QR determination in natural samples. The DTO/GC electrode was used for the first time in non-aqueous media. This modified electrode was found to be very sensitive to QR molecules and could be used for the determination of QR in the future studies. The next step in our study is to use this modified GC sensor electrode for the quantitative determination of QR using voltammetric techniques, and with this in mind, different plants are going to be collected and then varying antioxidant derivatives are going to be extracted from these plants. From the extract QR is going to be quantified by different voltammetric, spectroscopic and microscopic techniques (cyclic voltammetry, differential pulse voltammetry, square wave voltammetry, X-ray photoelectron spectroscopy, raman spectroscopy, ellipsometry, scanning electron microscopy, *etc.*) in these natural samples.
